# Diabetic Ketoacidosis Treatment Outcome and Associated Factors Among Adult Patients Admitted to the Emergency Department and Medical Wards at King Abdulaziz Medical City, Riyadh, Saudi Arabia

**DOI:** 10.7759/cureus.10067

**Published:** 2020-08-27

**Authors:** Abdulaziz Alotaibi, Abdulrahman Aldoukhi, Bayan Albdah, Jamila A Alonazi, Amjad S Alseraya, Najla Alrasheed

**Affiliations:** 1 Medicine, King Saud Bin Abdulaziz University for Health Sciences, Riyadh, SAU; 2 Biostatistics and Bioinformatics, King Abdullah International Medical Research Center, Riyadh, SAU; 3 Internal Medicine, King Abdulaziz Medical City, Riyadh, SAU

**Keywords:** diabetic ketoacidosis (dka), hospital stay, hospital mortality, treatment outcome, icu admission

## Abstract

Background

Diabetic ketoacidosis (DKA) is a life-threatening condition with high morbidity and mortality rates. It should be diagnosed immediately and managed intensively to prevent its significant complications.

Objectives

The aim of this study to assess DKA treatment outcome and associated factors among adult patients at King Abdulaziz Medical City Emergency Department and Medical Wards, Riyadh, Saudi Arabia.

Materials and Methods

A retrospective cross-sectional study was conducted using a chart review to assess DKA treatment outcome and associated factors. All patients who were admitted as DKA cases from September 2017 to August 2019 were selected by simple random sampling except those with incomplete charts or younger than 14 years. Data were entered and analyzed using SAS Version 9.4 (SAS Institute, Cary, NC, USA).

Results

A total of 223 reviewed charts were collected. The frequency of DKA recurrence in most of the patients was once per year (126 [56.5%]). The most common precipitating factor was inappropriate insulin therapy (104 [46.64%]). More than half of the patients (120 [53.81%]) got out of DKA management protocol within 24-72 hours with a hospital stay of less than or equal to five days. The mortality rate was 1.83%. Patients with two or more DKA episodes per year tended to be admitted to ICU more frequently than those with one episode (p=0.001). It was found that patients who had a duration of one to five years of diabetes mellitus were almost five times more likely to get out of DKA in more than 72 hours when compared with those who had a duration of more than five years (adjusted OR: 4.7; 95% CI: 1.34-16.60; p=0.01).

Conclusions

The findings of this study highlight that majority of DKA patients showed improvement and discharged with a very low mortality rate. Inappropriate insulin therapy was the most common precipitating factor; thus, educating diabetic patients about the complications of treatment non-compliance is an important part of management.

## Introduction

Diabetic ketoacidosis (DKA) is one of the life-threatening acute hyperglycemic complications of diabetes mellitus (DM) carrying high morbidity and mortality among type 1 diabetics and less commonly but recognized in type 2 diabetics. It is characterized by massive hyperglycemia, ketonemia, and acidosis [1]. It is most commonly precipitated by infections, non-compliance to insulin therapy, or first presentation of diabetes. Other factors include stressors such as cerebrovascular accidents, cardiac ischemia, trauma, and pancreatitis [1-3].

DKA can result in significant complications as volume depletion and severe acidosis may lead to cardiac arrest and acute kidney injury (AKI); thus, prompt diagnosis and initiation of intensive treatment protocol by experienced staff are important for successful management [3,4] followed by careful monitoring to prevent the occurrence of iatrogenic complications with insulin and fluid administration, such as hypoglycemia, hypokalemia, and cerebral edema. Appropriate early fluid therapy and insulin administration show a significantly better outcome and fewer recurrences. DKA can be successfully managed within 12-36 hours with appropriate treatment, with which the complications and mortality can be prevented and reduced effectively [5].

To address this, a few studies were conducted to draw the actual picture regarding the DKA outcome and their risk factors in Saudi Arabia [6,7]. Therefore, this study helps health care workers to build a uniform treatment protocol. The gained information will help also both patients and health care providers to know the most common reasons for DKA recurrence to prevent future episodes. Furthermore, the results of this study can be used as a database for future researches. Keeping the aforesaid facts in view, this study aims to assess DKA treatment outcome and associated factors among adult patients at King Abdulaziz Medical City (KAMC) Emergency Department and Medical Wards, Riyadh, Saudi Arabia.

## Materials and methods

This retrospective cross-sectional study used a chart review to assess DKA treatment outcome and associated factors among adult patients at KAMC Emergency Department and Medical Wards. KAMC is a tertiary hospital located in Riyadh, the capital city of Saudi Arabia. It has a bed capacity of 690 beds and provides all types of care to all National Guard soldiers and their families, starting from primary health care up to tertiary specialized care.

To determine the sample size, the following assumptions were considered in this study. According to a study was conducted in Ethiopia to assess the DKA outcome, 84.90% of the patients were discharged with improvement with 95% confidence level and 5% margin of error [8]. The sample size was calculated by using Raosoft software (http://www.raosoft.com/samplesize.html). Based on this, the final expected sample size was approximately 196 patients. All patients who were admitted as DKA cases from September 1, 2017, to August 31, 2019, were included in the study, whereas patients with incomplete chart or younger than 14 years were excluded.

Simple random sampling was used to collect the data for both independent and outcome variables from BESTcare by using a checklist that was adapted from a related previous study [8]. Data were collected by the co-authors and data collectors. The collected data were checked for accuracy and completeness daily by the principal investigator. To identify any potential problems, the checklist was tested before the actual data collection on 10% of the total sample size, and some modifications were considered accordingly. The checklist included socio-demographic characteristics, complications, comorbidities, precipitating factors, frequency, clinical presentation, biochemical tests, renal function, electrolyte, complete blood count, and management protocol of DKA patients. Then, the collected data were used for analysis to assess DKA treatment outcome and associated factors.

Data were presented as mean ± standard deviation. Frequencies and percentages were used to describe categorical variables. We used Fisher’s exact test or chi-square test for association between categorical variables, and the Wilcoxon two-sample test, Kruskal-Wallis test (for not normally distributed data), t-test, or one-way analysis of variance (ANOVA) test (for normally distributed data) for continuous variables. We examined outcome “time within which patients get out of DKA” predictors using multivariate logistic regression. The regression model included age, type of DM, duration of DM, treatment regimen for DM, and comorbidities such as hypertension, dyslipidemia, and chronic kidney disease (CKD). All statistical tests were considered significant at p<0.05. Data were analyzed using the statistical program SAS Version 9.4 (SAS Institute, Cary, NC, USA).

Ethical approval was obtained from the Institutional Review Board at King Abdullah International Medical Research Center.

## Results

Demographics and clinical characteristics

A total of 223 reviewed charts were collected from the BESTcare system, of which 106 (47.53%) were males and 117 (52.47%) were females, and almost 60.54% of them were in the age group of 14-30 years. Out of the reviewed patients, 166 (74.44%) had type 1 diabetes and 57 (25.56%) had type 2 diabetes. Majority (184 [82.51%]) of the patients were on insulin treatment, and the other patients were on either oral (13 [5.83%]), both oral and insulin (19 [8.52%]), or not on any medication (7 [3.14%]). More than half of the patients had DM for more than five years (125 [56.05%]). Although most of the patients had no other chronic comorbidities, hypertension and dyslipidemia were documented in 52 (23.32%) and 50 (22.42%) patients, respectively. Table 1 shows the demographics and clinical characteristics profile of all respondents.

**Table 1:** Demographics and clinical characteristics (n=223) DM, diabetes mellitus; ACS, acute coronary syndrome; CKD, chronic kidney disease; BA, bronchial asthma

Variable	N	(%)
Age of the patient		
14-30 years	135	60.54
30.1-40 years	25	11.21
40.1-50 years	16	7.17
50.1-60 years	19	8.52
>60 years	28	12.56
Sex of the patient		
Male	106	47.53
Female	117	52.47
Educational background		
Elementary school	11	4.93
Tertiary school	16	7.17
Bachelor’s degree	3	1.35
Master’s degree	1	0.45
Unknown	192	86.10
Marital status		
Single	116	52.02
Married	73	32.74
Widowed	4	1.79
Divorce	2	0.90
Unknown	28	12.56
Type of DM		
Type 1	166	74.44
Type 2	57	25.56
Duration of DM		
1-5 years	44	19.73
>5 years	125	56.05
Newly diagnosed	14	6.28
Not documented	40	17.94
Treatment regimen for DM		
Insulin	184	82.51
Oral	13	5.83
Both	19	8.52
Not on any medications	7	3.14
Comorbidity		
Hypertension		
No	171	76.68
Yes	52	23.32
Dyslipidemia		
No	173	77.58
Yes	50	22.42
Heart failure		
No	215	96.41
Yes	8	3.59
ACS		
No	217	97.31
Yes	6	2.69
CKD		
No	211	94.62
Yes	12	5.38
BA		
No	212	95.07
Yes	11	4.93

The frequency of recurrent DKA and precipitating factors

The frequency of DKA recurrence per year was assessed and showed that most of the patients had DKA once (126 [56.5%]), 61 (27.35%) had two episodes, and 36 (16.14%) had three or more episodes. Inappropriate insulin therapy was the most common precipitating factor in 104 (46.64%) of the patients, which was mainly due to poor insulin compliance. Infections such as urinary tract infection, gastroenteritis, and upper and lower respiratory tract infections were the second most common precipitating factors in 70 (31.39%) of the DKA cases. Other precipitating factors are shown in Table 2.

**Table 2:** The frequency of recurrent DKA and precipitating factors (n=223) DKA, diabetic ketoacidosis; AMI, acute coronary syndrome; CVA, cerebrovascular accident; SGLT2, sodium-glucose co-transporter-2; UTI, urinary tract infection; URTI, upper respiratory tract infection; LRTI, lower respiratory tract infection

Variable	N	(%)
Frequency of recurrent DKA per year		
Once	126	56.50
Twice	61	27.35
≥3	36	16.14
Precipitating factors		
Inappropriate insulin therapy	104	46.64
Infection (urinary tract infection, respiratory tract infection, acute gastroenteritis, skin infection, etc.)	70	31.39
AMI	2	0.90
Sepsis of any origin	4	1.79
Pancreatitis	2	0.90
CVA	1	0.45
Drugs (steroids, thiazides, pentamidine, second-generation anti-psychotics, cocaine, immune checkpoints inhibitors, SGLT2 inhibitors)	2	0.90
Emotional stress	3	1.35
Any recent surgical intervention (<1 month)	2	0.90
Acute renal failure	6	2.69
Inappropriate diet	8	3.59
First presentation of DM	10	4.48
Unknown/not documented	9	4.04
Reasons for discontinued insulin		
Lack of supplies	1	0.96
Fade up of daily injection/poor compliance/missed doses	68	65.38
Combination of reasons	8	7.69
Insulin pump not working	6	5.76
Not documented	21	20.19
Type of infection		
UTI	10	14.28
URTI	5	7.14
LRTI	2	2.85
Gastroenteritis	7	10
Unknown source of infection/not documented	45	64.28

Clinical presentation of patients who were admitted with DKA

DKA patients were most commonly presented with abdominal pain (143 [64.13%]) and vomiting (139 [62.33%]). In addition, nausea (89 [39.91%]), polyuria/polydipsia (42 [18.83%]), and malaise (31 [13.90%]) were reported fairly by DKA patients. On physical examination, 141 (63.23%) of admitted patients with DKA were found to be looking ill with dehydration signs such as dry mucous membrane, poor skin turgor, sunken eyes, tachycardia, and hypotension. Regarding vital signs, the mean body temperature was 36.8±0.48°C, pulse rate was 106.14±19.49 beats/min, and systolic and diastolic blood pressures were 119.99±17.15 mmHg and 67.88±12.56 mmHg, respectively. Table 3 shows the details regarding the clinical presentation of patients.

**Table 3:** : Symptoms and signs of patients who admitted with DKA (n=223) DKA, diabetic ketoacidosis; SOB, shortness of breath; LOC, loss of consciousness

Symptoms of DKA		
Variable	N	(%)
Polyuria/polydipsia		
No	181	81.17
Yes	42	18.83
Nausea		
No	134	60.09
Yes	89	39.91
Vomiting		
No	84	37.67
Yes	139	62.33
Weight loss		
No	216	96.86
Yes	7	3.14
Malaise		
No	192	86.10
Yes	31	13.90
Abdominal pain		
No	80	35.87
Yes	143	64.13
Anorexia		
No	203	91.03
Yes	20	8.97
SOB		
No	193	86.55
Yes	30	13.45
LOC		
No	206	92.38
Yes	17	7.62
Weakness		
No	211	94.62
Yes	12	5.38
Signs of DKA		
Looking ill		
No	82	36.77
Yes	141	63.23
Dehydration signs (dry mucous. poor skin turgor, sunken eyes, tachycardia, hypotension)		
No	82	36.77
Yes	141	63.23
Constitutional signs		
No	195	87.44
Yes	28	12.56
Kussmaul respiration		
No	212	95.07
Yes	11	4.93
Ketone breath		
No	220	98.65
Yes	3	1.35
Altered mental status		
No	199	89.24
Yes	24	10.76
Temperature (°C)	36.91±0.48	
Systolic blood pressure (mmHg)	119.99±17.15	
Diastolic blood pressure (mmHg)	67.88±12.56	
Pulse rate (beats/min)	106.14±19.49	

Laboratory values of admitted patients with DKA

The average levels of random blood glucose, serum potassium, serum bicarbonate, and white blood cell (WBC) count were 27.65±9.8 mmol/L, 4.94±0.85 mmol/L, 13±6.56 mEq/L, and 13.27±6.43 (x10^9^/L), respectively. Besides, the average calculated anion gap for the chart-reviewed patients was 26.79±6.45, and the average urine ketones level was 109±51.3 mg/dL. Table 4 shows the details of the laboratory results.

**Table 4:** Laboratory values of admitted patients with DKA (n=223) BUN, blood urea nitrogen; WBC, white blood cell; RBC, red blood cell; DKA, diabetic ketoacidosis

Variable	Mean ± SD
Random blood glucose level (mmol/L)	27.65±9.8
Serum creatinine (µmol/L)	130.13±102.43
Serum BUN (mmol/L)	7.3±5.83
Serum sodium (mmol/L)	132.42±5.43
Serum potassium (mmol/L)	4.94±0.85
Anion gap calculation (mmol/L)	26.79±6.45
Serum bicarbonate (mEq/L)	13±6.56
Urinary ketones (mg/dL)	109.95±51.3
Serum amylase (U/L)	60.61±100.58
WBC count (x10^9^/L)	13.27±6.43
RBC count (x10^12^/L)	4.97±0.78
Hemoglobin (g/L)	137.95±25.03
Platelets (x10^9^/L)	340.67±120.16

Management protocol and outcome of DKA patients

While the most commonly used type of fluid bolus was 0.9% normal saline (218 [97.76%]) with an average amount of 1±0.08 liters, fluid maintenance was mostly with 5% dextrose in water (DW5%) (173 [77.58%]). The rate of fluid maintenance in majority (160 [71.75%]) of the patients was 100-120 mL/hour. Regular insulin was the only type of insulin used in all DKA patients with an average administration rate of 5.56±2.23 U/hour in the first 24 hours. Most (208 [93.69%]) of the patients were repleted with intravenous potassium chloride. The majority of DKA patients got out of DKA within 24-72 hours (120 [53.81%]) and less than 24 hours (76 [34.08%]). Nearly three-quarters of patients 170 (76.23%) did not develop any complications with DKA; however, AKI and hypokalemia were the most common complications in 30 (13.45%) and 8 (3.59%) of the patients, respectively. Most (152 [68.16%]) of the patients were treated in the general medical wards without the need for intensive care unit (ICU) admission, whereas the others (71 [31.84%]) were admitted to ICU mainly due to either severity of the illness, comorbidities, and/or developed complications. Almost 97 (43.50%) and 85 (38.12%) of the patients had a hospital stay of one to two days and three to five days, respectively. Concerning the treatment outcome of DKA patients, only four (1.79) patients showed no improvement and died, whereas the other patients (219 [98.21%]) showed improvement and discharged. Table 5 shows the details of the management protocol and outcome of DKA patients.

**Table 5:** Management protocol of DKA patients (n=223) DW5%, dextrose 5% in water; NS, normal saline; RL, Ringers lactate; DKA, diabetic ketoacidosis; ICU, intensive care unit

Variable	N	(%)
Type of IV fluid bolus		
Did not receive bolus because of overload	2	0.90
DW5%	3	1.35
NS	218	97.76
Amount of IV fluid bolus (in liters) (mean ± SD)	1±0.08	
Type of fluid maintenance used in the management		
DW5%	173	77.58
NS	48	21.52
RL	2	0.90
Rate of fluid maintenance used in the management		
60-70 mL/h	8	3.59
70-100 mL/h	33	14.80
100-120 mL/h	160	71.75
>120 mL/h	21	9.42
Patient refused and signed against medical advice	1	0.45
Insulin administration rate in the first 24 hours (U/h)	5.56±2.23	
Regular	223	100.00
Time within which patients get out of DKA		
24 h	76	34.08
>24 h and <72 h	120	53.81
>72 h	27	12.11
Potassium replacement		
No	14	6.31
Yes	208	93.69
Complications of DKA		
No complication	170	76.23
Hypoglycemia	5	2.24
Hypokalemia	8	3.59
Non-anion gap hyperchloremic acidosis	3	1.35
Cerebral edema/brain injury	1	0.45
Acute respiratory distress syndrome	1	0.45
Acute renal failure	30	13.45
Cardiac arrest	3	1.35
Others	2	0.90
Admitted to ICU		
No	152	68.16
Yes	71	31.84
Hospital stay		
1-2 days	97	43.50
3-5 days	85	38.12
>5 days	41	18.39
General treatment outcome of DKA patients		
Shows improvement and discharged	219	98.21
Shows no improvement (died)	4	1.79

Comparison among admitted ICU patients and associated factors

Patients with a frequency of recurrent DKA two times or more tended to be admitted to ICU more frequently than those who had only one episode per year (p=0.001). In addition, patients who were admitted with Kussmaul sign had a propensity to be admitted to ICU than those who were not (p=0.005). In addition, those who showed altered mental status at the time of presentation were likely to be admitted to ICU (p=0.002). Regarding the vital signs, patients with an initial pulse rate of 113.42±18.68 beats/min tended to be admitted to the ICU when compared with those with an initial pulse rate of 102.65±18.95 beats/min (p≤0.0001). Regarding the initial lab results, patients who presented with an investigation lab result of anion gap calculation of 28.24±7.01 mmol/L (p-0.03), serum bicarbonate of 9.61±7.49 mEq/L (p≤0.0001), and WBC count 1of 6.72±6.02 x 10^9^/L (p≤0.0001) were more likely to be admitted to ICU than the other group (26.11±6.08 mmol/L, 14.6±5.4 mEq/L, and 11.67±5.98 x 10^9^/L, respectively). The time within which the patients got out of DKA was found to be longer (>24 hours) in ICU patients when compared with those who got a DKA free in less than 24 hours (p≤0.0001). Also, those patients who were admitted to the ICU appeared to have a prolonged hospital stay (>five days) when compared with those who only stayed for less than two days (p≤0.0001). Table 6 shows the details for the comparison of response among patients who were admitted to ICU.

**Table 6:** Comparison among admitted ICU patients and associated factors (n=223) DM, diabetes mellitus; DKA, diabetic ketoacidosis; BUN, blood urea nitrogen; WBC, white blood cell; RBC, red blood cell

Variable	No (n=152)	Yes (n=71)	p-Value
Socio-demographic characteristics			
Age of the patient			0.6546
14-30 years	94 61.84	41 57.75	
30.1-40 years	16 10.53	9 12.68	
40.1-50 years	12 7.89	4 5.63	
50.1-60 years	14 9.21	5 7.04	
>60 years	16 10.53	12 16.90	
Sex of the patient			0.1140
Male	78 51.32	28 39.44	
Female	74 48.68	43 60.56	
Type of DM			0.7445
Type 1	112 73.68	54 76.06	
Type 2	40 26.32	17 23.94	
Duration of DM			0.5496
1-5 years	29 19.08	15 21.13	
>5 years	85 55.92	40 56.34	
Newly diagnosed	12 7.89	2 2.82	
Not documented	26 17.11	14 19.72	
Treatment regimen for DM			0.9845
Insulin	124 81.58	60 84.51	
Oral	9 5.92	4 5.63	
Both	14 9.21	5 7.04	
Not on any medications	5 3.29	2 2.82	
Frequency of recurrent DKA per year			0.0013
Once	98 64.47	28 39.44	
Twice	36 23.68	25 35.21	
≥3	18 11.84	18 25.35	
Symptoms of DKA			
Polyuria/polydipsia			0.1411
No	119 78.29	62 87.32	
Yes	33 21.71	9 12.68	
Nausea			0.7696
No	90 59.21	44 61.97	
Yes	62 40.79	27 38.03	
Vomiting			0.4601
No	60 39.47	24 33.80	
Yes	92 60.53	47 66.20	
Weight loss			0.6822
No	148 97.37	68 95.77	
Yes	4 2.63	3 4.23	
Abdominal pain			0.7646
No	56 36.84	24 33.80	
Yes	96 63.16	47 66.20	
SOB			0.0901
No	136 89.47	57 80.28	
Yes	16 10.53	14 19.72	
LOC			0.061
No	144 94.74	62 87.32	
Yes	8 5.26	9 12.68	
Signs of DKA			
Dehydration signs			0.0751
No	62 40.79	20 28.17	
Yes	90 59.21	51 71.83	
Constitutional signs			0.1276
No	129 84.87	66 92.96	
Yes	23 15.13	5 7.04	
Kussmaul respiration			0.0053
No	149 98.03	63 88.73	
Yes	3 1.97	8 11.27	
Ketone breath			0.2385
No	151 99.34	69 97.18	
Yes	1 0.66	2 2.82	
Altered mental status			0.0018
No	143 94.08	56 78.87	
Yes	9 5.92	15 21.13	
Temperature	36.93±0.47	36.85±0.48	0.5509
Systolic blood pressure (mmHg)	120.36±17.77	119.18±15.84	0.7131
Diastolic blood pressure (mmHg)	67.8±12.06	68.07±13.64	0.8681
Pulse rate in (beats/min)	102.65±18.95	113.42±18.68	
Laboratory values of admitted patients with DKA			
Random blood glucose level (mmol/L)	26.88±9.17	29.31±10.9	0.1649
Serum creatinine (µmol/L)	129.12±111.89	132.31±79.13	0.0623
serum BUN (mmol/L)	7.2±5.55	7.53±6.41	0.8655
Serum sodium (mmol/L)	131.88±5.4	133.59±5.35	0.0086
Serum potassium (mmol/L)	4.84±0.8	5.15±0.93	0.0069
Anion gap calculation (mmol/L)	26.11±6.08	28.24±7.01	0.0287
Serum bicarbonate (mEq/L)	14.6±5.4	9.61±7.49	
Urinary ketones (mg/dl)	108.02±50.81	114.16±52.49	0.2713
Serum amylase (U/L)	63.91±119.99	54.32±45.47	0.5402
WBC count (x10^9^/L)	11.67±5.98	16.72±6.02	
RBC count (x10^12^/L)	5.01±0.73	4.89±0.87	0.4863
Hemoglobin (g/L)	139.74±22.32	134.11±29.83	0.3528
Platelets (x10^9^/L)	326.98±109.46	370.4±136.81	0.0141
Management outcome of DKA patients			
Time within which patients get out of DKA			
=24 h	70 46.05	6 8.45	
>24 h and <72 h	70 46.05	50 70.42	
>72 h	12 7.89	15 21.13	
Hospital stay			
1-2 days	91 59.87	6 8.45	
3-5 days	46 30.26	39 54.93	
>5 days	15 9.87	26 36.62	
General treatment outcome of DKA patients			0.0963
Shows improvement and discharged	151 99.34	68 95.77	
Shows no improvement (referred or died)	1 0.66	3 4.23	

Predictors of time within which the patient gets out of DKA

Different factors have been studied as determinants of time within which the patient gets out of DKA. It was found that patients who had a duration of one to five years of DM were almost five times more likely to get out of DKA in more than 72 hours when compared with those who had a duration of more than five years (adjusted OR: 4.7; 95% CI: 1.34-16.60; p=0.01). Patients who had a treatment regimen of both oral medication and insulin were likely four times to get free of DKA in more than 72 hours as compared with those only were on insulin (adjusted OR: 4.5; 95% CI: 0.97-21.15; p=0.05). Patients with CKD had a likelihood to get out of DKA in more than 72 hours by more than four times as compared with those who were not (adjusted OR: 4.0; 95% CI: 0.86-18.68; p=0.04). Table 7 shows the predictors of time within which the patients got out of DKA.

**Table 7:** Predictors of time within which the patient got out of DKA (n=223) DM, diabetes mellitus; CKD, chronic kidney disease; DKA, diabetic ketoacidosis

Independent variable	<72 h (n=196)	>72 h (n=27)	OR “univariate”	95% CI		p-Value	Adjusted OR “multivariate”	95% CI		p-Value
				Lower	Upper			Lower	Upper	
Age (ref: 14-30 years)	124 63.27	11 40.74								
30.1-40 years	22 11.22	3 11.11	1.537	0.397	5.958	0.5339	1.137	0.216	5.987	0.8792
40.1-50 years	13 6.63	3 11.11	2.601	0.642	10.535	0.1803	1.548	0.253	9.474	0.6365
50.1-60 years	17 8.67	2 7.41	1.326	0.271	6.501	0.7278	0.496	0.060	4.066	0.5136
>60 years	20 10.20	8 29.63	4.510	1.617	12.581	0.0040	0.816	0.113	5.914	0.8404
Type of DM (ref: type 1)	151 77.04	15 55.56								
Type 2 vs type 1	45 22.96	12 44.44	2.684	1.172	6.149	0.0195	0.693	0.149	3.210	0.6387
Duration of DM (ref: >5 years)	116 59.18	9 33.33								
1-5 years	37 18.88	7 25.93	2.438	0.849	7.001	0.0977	4.720	1.342	16.600	0.0156
Newly diagnosed	13 6.63	1 3.70	0.991	0.116	8.461	0.9937	1.708	0.113	25.758	0.6989
Not documented	30 15.31	10 37.04	4.297	1.603	11.517	0.0038	3.691	1.138	11.969	0.0296
Treatment regimen for DM (ref: insulin)	166 84.69	18 66.67								
Both (oral and insulin)	13 6.63	6 22.22	4.257	1.442	12.570	0.0087	4.523	0.967	21.155	0.0552
Not on any medications	6 3.06	1 3.70	1.537	0.175	13.491	0.6981	2.240	0.136	36.934	0.5728
Oral	11 5.61	2 7.41	1.677	0.344	8.167	0.5223	1.161	0.175	7.689	0.8769
Hypertension (ref: no)	158 80.61	13 48.15								
Yes vs no	38 19.39	14 51.85	4.478	1.945	10.310	0.0004	3.205	0.849	12.105	0.0858
Dyslipidemia (ref: no)	158 80.61	15 55.56								
Yes vs no	38 19.39	12 44.44	3.327	1.440	7.687	0.0049	2.050	0.628	6.691	0.2342
CKD (ref: no)	189 96.43	22 81.48								
Yes vs no	7 3.57	5 18.52	6.136	1.794	20.986	0.0038	4.005	0.861	18.632	0.0369

## Discussion

Majority (98.21%) of the admitted DKA patients showed improvement and discharged. This result is much higher than a study conducted at Adama University Hospital, where only 84.90% showed improvement and were discharged [8]. In this study, the mortality rate is 1.83%, which is relatively close to that reported in a study conducted at Chang Gung Memorial Hospital, where only 0.67% of the admitted patients with DKA showed no improvement and died [9]. The mortality rate in this study is considered relatively low as compared to other studies that were conducted in Zambia and Malaysia with a mortality rate of 16.66% and 17.6%, respectively [10,11]. Concerning the hospital stay, majority of the patients had a hospital stay of less than or equal to five days, which is considered less than a study that was conducted locally at King Fahad Medical City with an average hospital stay of seven days [12]. This difference in improvement, mortality, and length of hospital stay can be explained mainly by the advanced age, associated comorbidities of the patients, and the different treatment protocols that were provided by different medical institutions.

In this study, the frequency of DKA recurrence in majority of patients was found to be only one episode per year. This shows lower recurrence as compared with a study was conducted at Adama University Hospital, in which the recurrence was found to be two or more episodes per year in most (65%) of the patients [8]. It is well known worldwide that the most common precipitating factor for DKA is infection followed by inappropriate insulin therapy [2,8]. In contrast, the most common precipitating factor in our study is inappropriate insulin therapy (46.64%) due to poor compliance followed by infections (31.39%). Also, this was reported in other various studies that were conducted in Saudi Arabia, which showed a similar pattern in poor insulin compliance, which was the most common among patients with recurrent DKA admissions [12-15]. This variation in precipitating factors was attributed to the difference in population across the world as explained in an article review that was published by Kitabchi et al. [1]. Further researches are needed to find out the reason behind fewer recurrence of DKA that is precipitated by inappropriate insulin therapy as found in our study.

Concerning the clinical features of DKA patients, abdominal pain (69%) was reported among the patients in a study published in 2015 at KAMC [15]. Vomiting (61.6%) was reported in another local study that was conducted among DKA adults in a tertiary hospital in Riyadh, Saudi Arabia [14]. Polyurea (26.3%) and polydipsia (28.2%) were found as initial clinical presentations among patients. In addition, dehydration signs including hypotension and tachycardia were reported in 61.8% of the patients in a study conducted locally at King Fahad Medical City [12]. These clinical features are consistent with our results.

Nearly three-quarters of the patients who were admitted with DKA did not develop complications associated with the disease. Majority (13.45%) of our patients were complicated with AKI, which is considered low as compared to a study conducted in Bangladesh showing that 29.5% of their patients developed AKI [16]. This variation could be explained by the severity of dehydration and how early fluid replacement is initiated. We found that 3.59% of our patients were complicated with hypokalemia, which is consistent with various studies reporting the incidence of hypokalemia in DKA patients to range from 0% to 4% during various stages of DKA management [17,18]. This is explained by insulin administration and correction of acidosis and hyperosmolarity that drive potassium intracellularly, resulting in hypokalemia; thus, careful monitoring of potassium is an important aspect of management [19].

Most of the DKA patients in our study were treated in the general medical wards (68.16%), which is consistent with a study conducted at Auckland Hospital in which almost 70% did not need to be admitted to ICU [20]. Similarly, other studies showed that more than half of their DKA patients were treated in the general medical wards [21,22]. Similar to a Libyan study, we found that patients who were admitted to ICU either due to severity of the episode, development of acute complications, or having other comorbidities stayed significantly longer in hospital (>five days) when compared to those who were treated in the general medical wards (<two days) [21].

With appropriate treatment of DKA, patients are expected to be out of DKA, which is defined as glucose less than 200 mg/dL and at least two of the following: venous pH over 7.30, serum bicarbonate more than or equal to 15 mEq/L, or anion gap less than or equal to 12 mEq/L within 48 hours [23,5]. In KAMC, the treatment protocol is generally according to the recent American Diabetic Association guidelines for DKA management [24]. The majority of DKA patients in our study got out of DKA within 24-72 hours (53.81%) or less than 24 hours (34.08%). We found that patients who were already diagnosed with DM and being treated with both oral and insulin were more likely to be out of DKA in more than 72 hours when compared to those who were on insulin alone. This can be explained by the concept that patients who are on both oral and insulin therapy are likely to be type 2 diabetics with advanced or uncontrolled disease and thus higher rates of complications and comorbidities. We could not find studies that support this result; thus, further studies are needed to identify factors that play a role in a prolonged time to be out of DKA.

Each study has its limitations. A limitation of our study is that we did not have much data regarding the treatment protocol in detail due to a poor chart review in this part. Therefore, we could not assess the management and its associated factors. Another limitation is that our study was only limited to one hospital (KAMC) and not generalized to other hospitals in the Riyadh region.

## Conclusions

The findings of this study highlight that majority of DKA patients showed improvement and were discharged with a very low mortality rate. Most of the patients received their medical treatment in medical wards, with a hospital stay of less than or equal to five days. Our data suggest that the time within which the patient got out of DKA is 24-72 hours. The frequency of DKA recurrence was found to be only one episode per year and precipitated commonly by poor insulin therapy and infections. Further studies are needed to better understand the management and its associated factors.
